# Vitamin D and COVID-19: Narrative Review after 3 Years of Pandemic

**DOI:** 10.3390/nu14224907

**Published:** 2022-11-20

**Authors:** Emanuele Gotelli, Stefano Soldano, Elvis Hysa, Sabrina Paolino, Rosanna Campitiello, Carmen Pizzorni, Alberto Sulli, Vanessa Smith, Maurizio Cutolo

**Affiliations:** 1 Laboratory of Experimental Rheumatology and Academic Division of Clinical Rheumatology, Department of Internal Medicine and Specialties, University of Genova, IRCCS San Martino Polyclinic Hospital, 16132 Genova, Italy; 2 Department of Internal Medicine, Ghent University Hospital, 9000 Ghent, Belgium; 3 Department of Rheumatology, Ghent University Hospital, 9000 Ghent, Belgium; 4 Unit for Molecular Immunology and Inflammation, Vlaams Instituut voor Biotechnologie (VIB), Inflammation Research Center (IRC), 9000 Ghent, Belgium

**Keywords:** vitamin D, neuroendocrine immunology, intracrinology, COVID-19, inflammation

## Abstract

Active vitamin D [1,25(OH)_2_D_3_—calcitriol] is a secosteroid hormone whose receptor is expressed on all cells of the immune system. Vitamin D has a global anti-inflammatory effect and its role in the management of a SARS-CoV-2 infection has been investigated since the beginning of the COVID-19 pandemic. In this narrative review, the laboratory and clinical results of a vitamin D supplementation have been collected from both open-label and blinded randomized clinical trials. The results are generally in favor of the utility of maintaining the serum concentrations of calcifediol [25(OH)D_3_] at around 40 ng/mL and of the absolute usefulness of its supplementation in subjects with deficient serum levels. However, two very recent large-scale studies (one open-label, one placebo-controlled) have called into question the contribution of vitamin D to clinical practice in the era of COVID-19 vaccinations. The precise role of a vitamin D supplementation in the anti-COVID-19 armamentarium requires further investigations in light of the breakthrough which has been achieved with mass vaccinations.

## 1. Introduction

Active vitamin D [1,25(OH)_2_D_3_—calcitriol] is a fat-soluble hormone that exerts multiple biological properties (endocrine, paracrine and intracrine) in the human body [1]. The paracrine and intracrine functions of vitamin D have aroused great interest, in particular for the almost ubiquitous expression of the vitamin D receptor (VDR) by the cells of the immune system, supporting a role in the regulation of the acute and chronic inflammatory response [2].

In particular, the link between vitamin D and inflammation in course of respiratory infections has been studied for more than a century, starting from the clinical pieces of evidence of the antimicrobial activity exerted by vitamin D against *Mycobacterium tuberculosis* [3]. Recently, a robust meta-analysis of more than 1500 researches on this topic identified a vitamin D supplementation as a protective factor against acute airways infections, thanks to its immunomodulatory properties [4]. Vitamin D does not act directly against the most common respiratory viruses (i.e., influenza virus, rhinovirus and respiratory syncytial virus), but it globally reduces the expression and secretion of pro-inflammatory chemokines and cytokines [5,6].

Following the spread of the COVID-19 pandemic between the end of 2019 and the beginning of 2020, several investigations have been carried out regarding the correlation between vitamin D [calcifediol—25(OH)D_3_] serum concentrations and the course of COVID-19, in order to assess whether a vitamin D supplementation could be beneficial even against an SARS-CoV-2 infection [7].

As low 25(OH)D_3_ serum concentrations are common in COVID-19 patients and correlate with a worse prognosis of the disease, ad hoc studies have been performed to evaluate the clinical effects of a vitamin D supplementation in COVID-19 patients.

This narrative review is therefore structured in first part concerning the physiology of vitamin D and the interconnection between its immunological effects and the inflammatory response caused by SARS-CoV-2, while the second part is focused on randomized clinical trials (RCTs), regarding the effects of a vitamin D supplementation on COVID-19 (susceptibility, disease course and the impact on vaccinations).

## 2. Vitamin D Physiology

Vitamin D derives from foods (both of animal and vegetable origin) and for the most part (about 80%), from cutaneous 7-dehydrocholesterol, that is converted into pre-vitamin D_3_ by ultraviolet B sun rays with wavelength between 290 and 315 nm [8,9].

Pre-vitamin D_3_ belongs to the steroid family thanks to the cholesterol-derived sterane ring, formed by four condensed rings of carbon atoms (secosteroid) [10]. Pre-vitamin D_3_ undergoes thermal processes of isomerization to cholecalciferol and it is released by epidermal keratinocytes in the bloodstream, where it circulates bound to a vitamin D-binding protein (VBP); on the other side, cholecalciferol of a food origin is incorporated in chylomicrons, reaches the venous circulation through the lymph and binds to the VBP [11].

Cholecalciferol undergoes the first hydroxylation in the hepatocytes in position 25 by mitochondrial and microsomal enzymes (CYP27A1 or CYP2R1). Calcifediol, or 25(OH)D_3_, is the resulting metabolite and the form that is usually measured in the serum to determine vitamin D concentrations, due to its high availability and long half-life (about three weeks). Calcifediol is therefore hydroxylated in position 1 by CYP27B1 in the kidney: calcitriol, or 1,25(OH)_2_D_3_, is the active final form of vitamin D [11].

CYP27B1 is almost ubiquitously expressed in the human body. When synthesized by renal proximal tubular cells, calcitriol exerts endocrine activities, regulating calcium-phosphorus homeostasis. In the small intestine, it increases the absorption of calcium and phosphorus, in the skeleton promotes bone mineralization, upregulating an osteoclastic differentiation and downregulating the release of the parathyroid hormone and in the kidneys, it stimulates the reabsorption of calcium [12]. All these activities are possible thanks to the expression of VDR on the target cells. VDR belongs to the nuclear receptor superfamily and when it binds to calcitriol, it regulates rapid non-genomic and slower genomic effects [13].

The endocrine effects of vitamin D are due to the non-genomic interactions of VDR, which stimulates the activation of signaling molecules and the formation of second messengers, that phosphorylate target protein kinases, regulating the entrance of calcium in the cells through the Ca^2+^ channels [14].

The extra-osseus effects of vitamin D are due to a conformational change in VDR, which heterodimerizes with the retinoic acid X receptor and migrates into the cell nucleus, regulating the transcription of thousands of genes, involved in immunomodulation and cell growth differentiation [15].

At last, calcitriol is inactivated by CYP24A1 (hydroxylation in position 24), excreted in the bile and then eliminated via the feces, after an enterohepatic recirculation [8].

## 3. Vitamin D Immunomodulatory Effects and SARS-CoV-2 Inflammatory Response

SARS-CoV-2 and active vitamin D almost always exhibit opposing biological actions.

SARS-CoV-2 is a beta coronavirus that is transmitted by airway droplets from human to human and expresses surface spike proteins, which bind to several receptors of human cells (CD26, CD147 and CD209) with the type 2 angiotensin-converting enzyme (ACE2) as the main target [16]. ACE2 is highly expressed by respiratory, gastrointestinal and endothelial cells [17]. SARS-CoV-2 uses the S1 subunit of its receptor-binding domain (RBD) to bind ACE2 and the co-receptors, while the S2 subunit is used to invade the target cells. Viral RNA nucleic acid is then replicated and exocytosed by the host cells to spread the infection [18].

ACE2 is not only a receptor for SARS-CoV-2 but mainly a key regulator enzyme of the renin–angiotensin–aldosterone system. ACE2 catalyzes the conversion of angiotensin II to angiotensin 1–7, which promotes vasodilatory, anti-inflammatory and antithrombotic effects, by acting on the AT2 and MAS receptors [19]. ACE2 also counteracts the action of the ACE enzyme, which, on the contrary, favors the production of angiotensin II, that promotes the increase in the peripheral vascular resistance, endothelial pro-coagulative dysfunction and pulmonary interstitial fibrosis, by acting on the AT1 receptors [19]. SARS-CoV-2 causes an imbalance of the ACE2/ACE ratio, favoring the detrimental biological effects of ACE [17].

On the contrary, calcitriol stimulates the expression of ACE2 in different human tissues and helps to restore a physiological ACE2/ACE ratio, in opposition to the viral prothrombotic and proinflammatory effects [20].

### 3.1. Innate Immunity Activation in COVID-19

SARS-CoV-2 usually infects cells in the upper respiratory tract, triggering the first line of defense, represented by innate immunity. SARS-CoV-2 is able to overcome the mucus produced by epithelial cells and the defense proteins contained in it, activating cellular pattern recognition receptors (PRRs) and consequently the innate immune response [21].

Among PRRs, SARS-CoV-2 is usually recognized by Toll-Like Receptor (TLR)-2 and TLR-4, expressed on the surface of immune (monocytes, macrophages and dendritic cells), endothelial and epithelial cells [22]. The stimulation of TLR-2 and TLR-4 signaling causes the release of pro-inflammatory cytokines, such as interleukin (IL)-1β, IL-6, IL-8, IL-17, IL-18, IL-33 and tumor necrosis factor (TNF)-α, mediated by Nod-like receptor protein 3 (NLRP3) inflammasome and NF-kB transcription factor [23].

Moreover, TLR-4 can activate the type I interferon (IFN) antiviral pathway through the downstream/adaptor proteins TRIF (TIR-domain-containing adapter inducing IFN-β), TRAF3 (TNF receptor-associated factor 3) and IRF3 (interferon regulatory factor 3) [24]. However, SARS-CoV-2 downregulates the production of type I IFN, blocking the cyclic GMP-AMP synthase (cGAS)-stimulator of interferon genes (STING), which acts as an activator of IRF3 [24].

Interestingly, calcitriol attenuates TLR-2 signaling as well as the activation of the NLRP3 inflammasome/NF-kB axis in animal and human models of inflammatory and autoimmune diseases (rheumatoid arthritis and systemic lupus erythematosus) [25,26,27,28].

After a recognition by PRRs, SARS-CoV-2 is able to escape defense mechanisms, in particular the autophagy process [29]. SARS-CoV-2 downregulates the autophagy promoters, such as the mechanistic target of rapamycin complex 1 (mTORC1) and AMP-activated protein kinase (AMPK) activator pathways, and upregulates autophagy inhibitors, such as RAC-alpha serine/threonine-protein kinase (AKT1) and S-phase kinase-associated protein 2 (SKP2) [30]. Moreover, the fusion between autophagosomes and lysosomes is impaired by the virus, due to the degradation of autophagy-initiating protein Beclin-1 (BECN-1) [31]. SARS-CoV-2 also degrades TANK-binding kinase 1 (TBK1), that regulates not only an autophagy initiation but also the production of type I IFN [32].

It is of note that calcitriol hinders the replication of SARS-CoV-2 at the intracellular level, inducing the expression of BECN-1, so promoting autophagy [33,34].

The viral activation of PRRs then causes the recruitment of innate immunity cells, in particular neutrophils, monocytes/macrophages and dendritic cells.

Neutrophils are recruited in affected tissues by IL-8 and they release extracellular traps (NETs) of nuclear material to bind and destroy pathogens [35]. Animal models of COVID-19 have suggested that paradoxically H3 and H4 histones released with NETosis increase the infectious capacity of SARS-CoV-2 rather than counter it [36]. Furthermore, inefficient NETs favor the formation of microthrombi in damaged tissues, a peculiar feature of the endothelial injury caused by the virus [37].

Calcitriol can reduce the expression of NETs in rat models of pulmonary diseases [38]. Analogously, it stimulates the release of cathelicidin by neutrophils and the LL-37 peptide which promotes, through macrophages, the removal of NETs and hinders the binding between SARS-CoV-2 subunits and ACE2 receptors [39,40]. Indeed, LL-37 peptide and the total number of serum leukocytes ratio in COVID-19 patients has been correlated with the severity of the disease [41].

Monocytes can be directly infected by SARS-CoV-2, through Fcγ receptors, stimulating NLRP3 inflammasome together with other pro-inflammatory mediators [42,43]. The final result is a classical activation of macrophages (M1), that release the aforementioned pro-inflammatory cytokines. It is of note that calcitriol induces the expression of IL-10 by immune cells, so promoting an alternative/anti-inflammatory activation of macrophages (M2) [28].

At last, the dendritic cells’ production of type I IFN is impaired, as well as the release of IL-12 and IL-23, resulting in a reduced activation of the T helper (Th)-17 response [44]. In this case, the biological effects of calcitriol seem superimposable and less advantageous, as it too reduces the production of IL-12 and IL-23, favoring a more tolerogenic state [45].

### 3.2. Adaptive Immunity Response in COVID-19

When the defense mechanisms of innate immunity are not able to resolve the infection, monocytes, macrophages and dendritic cells present SARS-CoV-2 antigen peptides complexed with the major histocompatibility complex class II to naïve T cells [46]. The activation of CD4+ T cells is predominant compared to CD8+ T cells, a ratio that remains constant even with the latest variants of the virus [47]. T helper (Th)-1 cells release type II IFN (or IFN γ) and TNF, which activate the cell-mediated response, polarizing macrophages towards a pro-inflammatory M1 phenotype [47]. These T-cells are usually effective in resolving a SARS-CoV-2 infection and are stimulated by anti-COVID-19 vaccines [48]. However, in the most severe cases of COVID-19, the inflammatory response is unable to eliminate SARS-CoV-2 and the impairment of T regulator cells together with the intense Th1 activation which manifests itself with the peripheral blood lymphopenia and subsequently with an inflammatory abnormal response that can lead to the notorious “cytokine storm”, with dramatic clinical manifestations, such as acute respiratory distress syndrome [49,50].

On the other hand, calcitriol down-regulates the Th1 response, activating several transcription factors in CD4+ T cells that shift the production of cytokines towards IL-10, which has an anti-inflammatory effect, polarizing also the macrophages toward an anti-inflammatory M2 phenotype [51].

The production of anti-SARS-CoV-2 antibodies develops from naïve B cells and begins a few days after the onset of COVID-19 symptoms [47]. Immunoglobulins (Ig)M are directed against viral nucleocapsid, spike proteins and RBD, the latter with neutralizing properties [47]. Seroconversion into IgG is completed after ten days [47]. IgA are also crucial for COVID-19 patients: they are secreted by mucosa-associated lymphoid tissue and promotes viral shedding from the airway’s epithelium [52].

Interestingly, calcitriol promotes the differentiation of naïve B cells towards IgA-secreting plasmablasts, with an active role in the defense mucosal [53].

**Figure 1:**
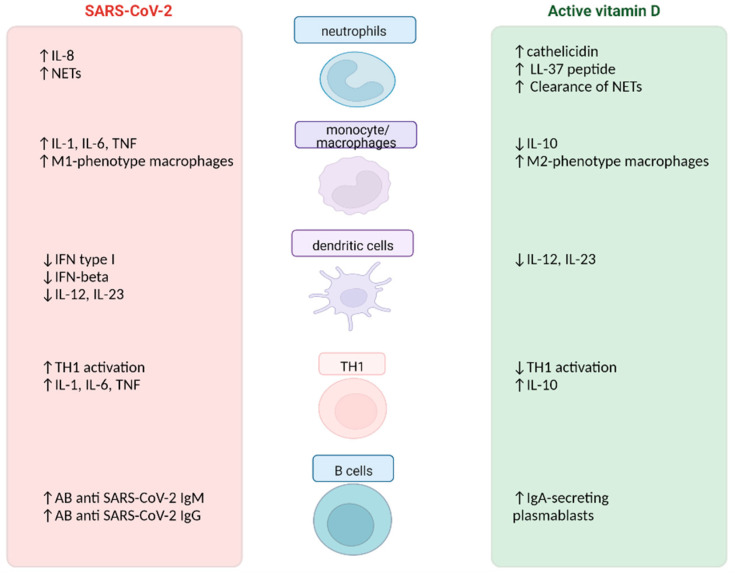
Main biological effects of SARS-CoV-2 and active vitamin D on immune cells. Abbreviations: AB: antibodies, Ig: immunoglobulin; IFN: interferon; IL: interleukin; TH: T helper; TNF: tumor necrosis factor; M1: classically activated macrophages; M2: alternatively activated macrophages; NETs: neutrophil extracellular traps. Symbols: ↑: upregulation; ↓: downregulation. Produced at www.biorender.com (accessed on 11 November 2022).

The schematic model reported so far of an acute SARS-CoV-2 infection derives for the most part from experiences regarding the first circulating variants of the virus (alpha–delta) [54]. The emergence of ever new viral variants, due to nucleotide changes in the SARS-CoV-2 genome, caused by RNA replication errors with consequent mutations in host cell binding proteins, does not seem to significantly modify the biological interactions with vitamin D [54]. In fact, active vitamin D shows global anti-inflammatory effects, summarized in Figure 1, which may mitigate the inflammatory response induced by SARS-CoV-2, but that are certainly not anti-SARS-CoV-2 specific [54].

## 4. Vitamin D Serum Concentrations and COVID-19

### 4.1. Effects of Vitamin D on Susceptibility to COVID-19

In recent years, 25(OH)D_3_ serum concentrations, especially below 25 nmol/L (10 ng/mL) have been identified as a risk factor for susceptibility to viral respiratory infections [55]. As a consequence, several studies have been performed to investigate the correlation between 25(OH)D_3_ serum concentrations and the susceptibility to SARS-CoV-2 and a recent meta-analysis of fifty-four papers has shown that a 25(OH)D_3_ deficiency (less than 30 ng/mL) was significantly associated with a SARS-CoV-2 infection (odds ratios between 1.49 and 1.83 depending on the levels of 25(OH)D_3_ deficiency) [56]. Of note, an observational study of 379 United Kingdom (UK) healthcare workers has found a U-shaped relationship between 25(OH)D_3_ serum concentrations and SARS-CoV-2 seropositivity: the susceptibility to COVID-19 increases with 25(OH)D_3_ serum concentrations below 30 ng/mL (the lower the levels, the greater the risk), but, surprisingly, even with 25(OH)D_3_ serum concentrations above 40 ng/mL (the higher the levels, the greater the risk) [57]. Therefore, 25(OH)D_3_ serum concentrations of 40 ng/mL seem the optimal target in the general population [7]. However, to explain the increase in the infectious risk reported in the previous study, it is important to remember that reaching high 25(OH)D_3_ serum concentrations too quickly is counterproductive, as they activate fibroblast growth factor-23 (FGF-23) and 24-hydroxylase signaling, which inactivates calcitriol [58,59].

### 4.2. Effects of Vitamin D on Severity of COVID-19

Several studies have investigated the correlation between 25(OH)D_3_ serum concentrations and the severity of a SARS-CoV-2 infection (disease duration, pulmonary involvement, risk of need for intensive care units—ICUs—and overall mortality) [60]. 25(OH)D_3_ significantly correlates with the length of hospitalization, the need for invasive cares, such as mechanical ventilation, the lung involvement and the mortality [61,62,63,64,65,66,67,68,69]. Although there is not a total agreement in the observational studies conducted so far [70,71], most of the meta-analyzes confirm the significant correlation between 25(OH)D_3_ serum concentrations and the severity of COVID-19, even when caused by the more recent omicron subvariants of SARS-CoV-2 [72,73,74,75,76].

### 4.3. COVID-19 and Effects of Vitamin D Supplementation

In light of the previously reported evidence, firstly open-label and subsequently placebo-controlled RCTs evaluated the efficacy of a vitamin D supplementation in reducing the impact of COVID-19 [77]. Some authors were concerned that low 25(OH)D_3_ serum concentrations found in COVID-19 patients could be interpreted more as a consequence of the systemic inflammation, rather than a predisposing factor for the development of the disease [78,79].

The evidence from open label and single-blinded RCTs was immediately encouraging. In fact, even in the presence of different prescriptive schemes (i.e., 0.266–0.532 mg of oral calcifediol three times for the first week of the disease and then weekly, 0.5 mcg of calcitriol per day for two weeks, 1000–2000 IU of cholecalciferol for 7–14 days, 5000 IU of cholecalciferol per day for two weeks, 10,000 IU of cholecalciferol per day for two weeks, 50,000 IU of cholecalciferol on the first and eighth day of hospitalization or 400,000 IU of oral cholecalciferol within 72 h after COVID-19 diagnosis), a vitamin D supplementation was associated with a reduction in inflammatory markers (IL-6), an improvement in the lung functions (arterial oxygen saturation/inspired fraction of oxygen ratio) and a reduction in hospitalization, access to ICUs and the mortality rate of COVID-19 patients [80,81,82,83,84,85,86,87]. However, in a large open-label RCT regarding 6200 adults in the UK (CORONAVIT Study), 800 IU per day or 3200 IU per day of cholecalciferol for six months were not able to reduce the risk of acquiring SARS-CoV-2 in healthy volunteers, in comparison to a control group [88].

Furthermore, also placebo-controlled RCTs have been designed and conducted, providing conflicting data (the results are resumed in Table 1).

On the one hand, there were studies in favor of a vitamin D supplementation. Oral calcifediol, equivalent to 3000 to 6000 IU of cholecalciferol per day for two months, significantly decreased the peripheral neutrophil-to-lymphocyte ratio in COVID-19 patients, a functional parameter associated with a reduction in the access to ICUs and mortality [89]. Similarly, oral cholecalciferol (60,000 IU daily for a week) significantly accelerated the healing, decreasing the SARS-CoV-2 RNA in infected patients [90]. Moreover, 25,000 IU of cholecalciferol for four consecutive days, followed by 25,000 IU weekly for up to six weeks significantly improved the clinical conditions of COVID-19 patients reducing the request of an oxygen supplementation and the length of their hospital stay [91]. At last, a supplementation of 4000 IU daily of cholecalciferol for 30 days significantly decreased the risk of suffering from a SARS-CoV-2 infection [92].

However, other placebo-controlled RCTs questioned the usefulness of a vitamin D supplementation. For example, a single high dose of 200,000 IU of cholecalciferol proved ineffective to reduce the rate of ICUs access or the global mortality of COVID-19 hospitalized patients [93]. A similar conclusion was obtained with a single supplementation of 500,000 IU of oral cholecalciferol [94]. Although these results were predictable, due to the negative effects of FGF-23 and 24-hydroxylase, activated by single high doses of vitamin D, another more recent study was disappointing [95]. A total of 17,278 adults were supplemented with 5 mL/day of cod liver oil (containing approximately 400 IU of cholecalciferol) for up to six months in Norway: no difference was found in COVID-19 incidence and disease course in comparison with a placebo group (17,323 adults) [95].

Taken together those studies suggest that a vitamin D supplementation is efficient in COVID-19 when administered for a medium or long term, whereas high and/or single doses were found not to be effective.

### 4.4. Effects of Vitamin D Supplementation in COVID-19 Vaccinations

The development of anti-COVID-19 vaccines has turned the fight against SARS-CoV-2 and its variants in a positive way [96]. It has been hypothesized that vitamin D may positively influence the efficacy of vaccines, considering that low vitamin D serum concentrations were associated with an insufficient humoral response after a COVID-19 vaccinations in patients suffering from solid tumors [97,98]. However, a sub analysis of the recent aforementioned open-label CORONAVIT study found no efficacy from a supplementation of 800 or 3200 IU of cholecalciferol daily for 6 months in improving the immunogenicity of anti-COVID-19 vaccines ChAdOx1 nCoV-19 and BNT162b2 [99].

## 5. Conclusions

The solid pathophysiological rationale on the usefulness of a vitamin D supplementation also in a SARS-CoV-2 infection has not been fully endorsed by the current RCTs, due to the well-known difficulties in designing clinical studies on this topic for multiple variables (i.e., the baseline characteristics of the treated population, vitamin D supplementation regimens and different outcomes) [100].

However, most of the evidence gathered so far confirms the biological and clinical utility of a vitamin D supplementation in COVID-19 patients, in particular when the 25(OH)D_3_ serum concentrations are deficient. Current and future RCTs should clarify the most advantageous dosage of a vitamin D supplementation and the target population that can benefit the most from it.

At last, it needs to be definitively clarified whether a vitamin D supplementation can also be recommended for a subject vaccinated against COVID-19. In fact, unlike RCTs performed in the pre-vaccination period, the most recent RCTs seem to overshadow the role of a vitamin D supplementation when taken together with COVID-19 vaccines [95,99]. This challenging question is still open at the moment.

## Figures and Tables

**Table 1:** Randomized double-blind, placebo controlled clinical trials regarding the biological and clinical effects of vitamin D supplementation in COVID-19 prevention and treatment.

Trials	Study Population	Patients’ Cohorts’ Characteristics	Recruitment Period	Time of Follow-Up	Supplementation Regimen	Effects of Vitamin D Supplementation
Treatment with 25-hydroxyvitamin D_3_ (calcifediol) is associated with a reduction in the blood neutrophil-to-lymphocyte ratio marker of disease severity in hospitalized patients with COVID-19: a pilot multicenter, randomized, placebo-controlled, double-blinded clinical trial (Maghbooli Z et al., 2021, Ref. [89])	106 COVID-19 adult hospitalized patients with 25(OH)D_3_ serum concentrations < 30 ng/mL	53 patients on vitamin D_3_ group 53 patients on placebo group	May 2020–October 2020	2 months	25 mcg of 25(OH)D_3_ daily (equivalent to 3000–6000 IU of cholecalciferol) in addition to standard care	Increase in neutrophils to lymphocytes ratio
Short term, high-dose vitamin D supplementation for COVID-19 disease: a randomized, placebo-controlled, study (SHADE study) (Rastogi A et al. 2022, Ref. [90])	40 COVID-19 hospitalized patients with mild symptoms or asymptomatic	16 patients with 25(OH)D serum concentrations < 20 ng/mL received vitamin D_3_ treatment 24 patients received placebo	2020	21 days	60,000 IU daily of cholecalciferol (oral nano-liquid droplets) for a week in addition to standard care. If 25(OH)D serum concentrations were < 50 ng/mL in the treatment group, supplementation was continued for another week	Faster healing Decrease in serum fibrinogen
Positive effects of vitamin D supplementation in patients hospitalized for COVID-19: a randomized, double-blind, placebo-controlled trial (De Niet S et al., 2022, Ref. [91])	50 COVID-19 hospitalized patients with 25(OH)D_3_ serum concentrations < 20 ng/mL	26 patients received vitamin D_3_ supplementation 24 patients received placebo	August 2020–August 2021	9 weeks	25,000 IU daily of cholecalciferol over four consecutive days followed by 25,000 IU weekly of cholecalciferol in addition to best available treatment	Decrease in length of hospital stay Decrease in duration of supplemental oxygen request Improve of clinical recovery, assessed by WHO scale
Efficacy and safety of vitamin D supplementation to prevent COVID-19 in frontline healthcare workers. A randomized clinical trial. (Villasis-Keever. et al., 2022, Ref. [92])	321 SARS-CoV-2 free healthcare workers not receiving vitamin D supplementation	160 healthcare workers received vitamin D supplementation 161 healthcare workers received placebo	15 July 2020–30 December 2020	30 days	4000 IU daily of cholecalciferol capsules	Lower infection rate without serious adverse events
Effect of a single high dose of vitamin D3 on hospital length of stay in patients with moderate to severe COVID-19: a randomized clinical trial (Murai IH et al., 2021, Ref. [93])	240 COVID-19 adult hospitalized patients	120 patients received vitamin D supplementation 120 patients received placebo	2 June 2020–7 October 2020	Hospitalization period	200,000 IU of cholecalciferol in a single oral dose	No effects on in-hospital mortality, admission to intensive care unit or need for mechanical ventilation
High-dose vitamin D versus placebo to prevent complications in COVID-19 patients; multicentre randomized controlled clinical trial (Mariani J et al. 2022, Ref. [94])	218 COVID-19 adult hospitalized patients	115 patients received vitamin D_3_ supplementation 103 patients received placebo	14 August 2020–22 June 2021	Hospitalization period	500,000 IU of oral cholecalciferol (5 capsules of 100,000 IU) in a single oral dose	No change in the respiratory Sepsis related Organ Failure Assessment (SOFA) score between baseline and the highest value recorded up to day 7 No difference for length of hospital stays, intensive care unit admissions and in-hospital mortality
Prevention of COVID-19 and other acute respiratory infections with cod liver oil supplementation, a low dose vitamin D supplement: quadruple blinded, randomised placebo controlled trial (Brunvoll SH et al., 2017, Ref. [95])	34,601 adults not receiving vitamin D supplementation	17,278 adults received cod liver oil 17,323 adults received placebo	10 November 2020–2 June 2021	6 months	400 IU daily of cholecalciferol	No decrease in the incidence of SARS-CoV-2 infection and serious COVID-19 (self-reported dyspnoea, admission to hospital, death)

## Data Availability

No new data was provided in this manuscript.
